# Dual Spectral Matching in Perovskite Solar Cells via Upconverting plus Downshifting Nanoparticles

**DOI:** 10.1021/acsaem.5c02611

**Published:** 2025-09-30

**Authors:** Milliane P. S. Palácio, Luis P. M. dos Santos, Leonardo C. E. Barros, Nagyla Oliveira, Sergio F. N. Coelho, Edgar A. C. Coimbra, Daniel P. Camilo, F. Anderson S. Lima, Fernando E. Maturi, Ugur D. Menda, Fernando A. Sigoli, Wagner F. Silva, Carlos Jacinto, Paulo Andre, Manuel J. Mendes, Rute A. S. Ferreira, Igor F. Vasconcelos

**Affiliations:** † Department of Metallurgical and Materials Engineering, Federal University of Ceará, Fortaleza, Ceará 60455-760, Brazil; ‡ Department of Physics, Federal University of Alagoas, Maceió, Alagoas 57072-900, Brazil; ¶ Department of Inorganic Chemistry, State University of Campinas, Campinas, São Paulo 13083-970, Brazil; § i3N/CENIMAT, Department of Materials Science, NOVA School of Science and Technology and CEMOP/UNINOVA, Campus de Caparica, Caparica 2829-516, Portugal; ∥ Center of Technology, University of Fortaleza, Fortaleza, Ceará 60811-905, Brazil; ⊥ Department of Physics and CICECO - Aveiro Institute of Materials, University of Aveiro, Aveiro 3810-193, Portugal; # Department of Electrical and Computer Engineering and Instituto de Telecomunicações, Instituto Superior Técnico, Universidade de Lisboa, Lisbon 1049-001, Portugal

**Keywords:** Upconversion, downshifting, nanoparticles, lanthanides, perovskite solar cells

## Abstract

Maximizing solar cell efficiency is a critical step toward revolutionizing photovoltaic technologies and harnessing the full potential of light for energy conversion. This study investigates the incorporation of NaGdF_4_:Yb^3+^,Tm^3+^@NaGdF_4_:Eu^3+^ (TMN) and NaGdF_4_:Yb^3+^,Er^3+^@NaGdF_4_:Eu^3+^ (ERN) nanoparticles into perovskite solar cells (PSCs) to improve their power conversion efficiency. The nanoparticles were synthesized through thermolysis and characterized using multiple techniques, including photoluminescence spectroscopy, quantum yield measurements, transmission electron microscopy, X-ray diffraction, solar simulation, and external quantum efficiency assessments. These lanthanide-doped NPs exhibited strong downshifting and minor upconversion luminescence, acting like optical translators that reshape poorly absorbed light into usable wavelengths. Devices incorporating the ERN nanoparticles demonstrated a 22.19% relative increase in power conversion efficiency, while those with TMN showed a 13.23% improvement. These enhancements are attributed mainly to the effective downshifting emission of Eu^3+^ and improved surface passivation from the core–shell architecture, which together reduce recombination losses and improve charge carrier dynamics. These findings underscore the potential of photon-converting lanthanide-based materials to address spectral absorption limitations in PSCs, offering a promising route toward next-generation photovoltaic technologies.

## Graphical abstract

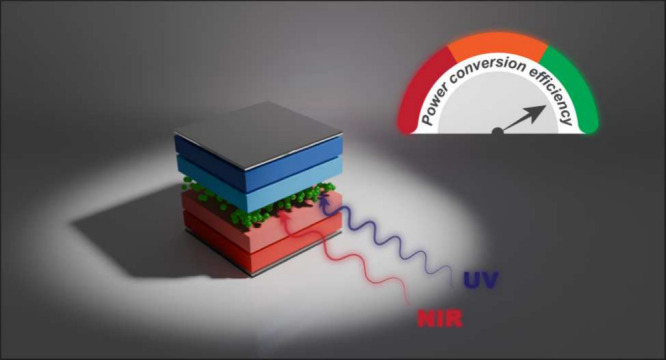

## 1 Introduction

The increasing global demand for energy, coupled with the urgent need to reduce greenhouse gas emissions and mitigate the impacts of climate change, has driven a global shift toward clean and sustainable energy sources. Among these alternatives, photovoltaic technologies, which directly convert sunlight into electricity, have emerged as a promising solution to address contemporary energy challenges. About 173,000 TW of energy hit the surface of Earth (land and water) continuously.^1^ This energy is enough to supply the World’s total energy consumption. According to the International Energy Agency (IEA), global electricity generation in 2024 amounted to approximately 30.9 TWh, reflecting continued growth in energy demand.^2^ In this context, perovskite solar cells (PSCs) have gained significant attention as one of the most innovative technologies, offering high power conversion efficiency (PCE), exhibiting a record PCE of 28.4%.^3^ In addition to high efficiency, PSCs offer cost-effective production, a wide range of applications, such as flexible substrates and portable devices, and exceptional performance under low-light environments, such as cloudy weather. These advantages make PSCs a viable option to expand the global solar energy market, presenting a promising pathway to meet the growing demand for sustainable and affordable electricity.^4,5^

Despite their rapid advancements, perovskite solar cells face significant challenges, including long-term stability issues exacerbated by photoinduced degradation and the limited spectral absorption range of perovskite materials.^6^ This limitation hinders their ability to efficiently capture low-energy photons, particularly those with energies below the bandgap of the active material, resulting in substantial energy losses and restricting the maximum performance potential of PSCs. Addressing these challenges—improving spectral absorption, mitigating photoinduced degradation, and enhancing long-term stability—is essential for boosting conversion efficiency and ensuring the viability of this technology for large-scale deployment.^7,8^

One promising approach to tackle these issues is the incorporation of lanthanide-doped NPs in PSCs, which can significantly improve device performance. Owing to their unique electronic structure characterized by partially filled 4*f* orbitals, these nanomaterials can convert photons from the near-infrared and ultraviolet regions of the electromagnetic spectrum into the visible range.^9^

Consequently, the observed luminescence results from transitions within these 4*f* orbitals, which, although formally forbidden by selection rules, enable photon conversion processes, including upconversion, downconversion, and downshifting, despite their typically low quantum efficiencies.

It is well established that luminescence processes in trivalent lanthanide-based materials are characterized by sharp emission lines and typical lifetimes ranging from microseconds to milliseconds, depending on the host matrix and environment.^9,10^ These features make lanthanides particularly suitable for photon-management applications, where prolonged emission and tunable spectra are key features, usually observed in the millisecond range. Such characteristics highlight the ability of lanthanides to enhance the optical performance of optoelectronic devices, including PSCs.^9^

By incorporating lanthanide-doped materials into solar cells, photons from nonabsorbed regions of the electromagnetic spectrum (e.g., infrared) can be converted into visible photons, potentially enhancing the spectral response. Additionally, their long decay times facilitate exciton dissociation while minimizing recombination losses. These advantages underscore the potential of lanthanides to drive the development of next-generation photovoltaic technologies.^9^

For instance, NPs such as NaGdF_4_:Yb^3+^/Er^3+^ and core–shell NaGdF_4_:Yb^3+^, Er^3+^@NaGdF_4_:Eu^3+^ have demonstrated improvements in the power conversion efficiency of perovskite solar cells by enhancing photon absorption through upconversion, achieving PCEs of up to 21.10% and 14.21%, respectively.^11,12^ Similarly, the integration of erbium-doped NPs with carbon quantum dots has led to an increase in PCE from 16.65% to 18.15%, while YLiF_4_:Yb^3+^,Er^3+^ NPs have boosted PSC performance from 19.00% to 21.32%.^13,14^ Hybrid NPs, such as NaCsWO_3_@NaYF_4_: Yb^3+^,Er^3+^ and europium-based compounds, have also been employed to improve spectral response, stability, and interfacial modification, further enhancing PCEs to impressive levels of 18.89% and 19.07%, respectively.^15,16^ Additionally, core–shell β-NaYF_4_:Yb^3+^,Tm^3+^@TiO_2_ upconversion NPs integrated into the mesoporous TiO_2_ layer achieved a 16.38% enhancement in efficiency, resulting in a PCE of 16.27%.^15,17^

In this study, we investigate the incorporation of two types of core–shell nanoparticles, NaGdF_4_:Yb^3+^,Tm^3+^@NaGdF_4_:Eu^3+^ (TMN) and NaGdF_4_:Yb^3+^,Er^3+^@NaGdF_4_:Eu^3+^ (ERN), as an interfacial layer in PSCs. These nanostructures exhibit dual spectral conversion capabilities, enabling simultaneous upconversion (via Yb^3+^/Tm^3+^ or Yb^3+^/Er^3+^ in the core) and downshifting (via Eu^3+^ in the shell), effectively extending the spectral response of the device. Although photon-converting nanomaterials have been explored in PSCs, the simultaneous and spatially selective doping of distinct lanthanide ions into the core and shell is uncommon, representing a key innovation of this work.

Additionally, the nanoparticles were synthesized through a high-temperature and high-pressure thermal decomposition route, which ensures high crystallinity and phase purity—critical for achieving efficient luminescent behavior. Uniquely, the NPs were incorporated into the PSCs without ligand removal or sintering, meaning their surface remained coated with native organic ligands (e.g., oleic acid). While such surface ligands are typically removed to avoid charge transport issues, here they were retained, simplifying processing and highlighting the dominant role of the nanoparticles’ optical effects. This strategy demonstrates that even in the presence of insulating surface groups, the photon-converting properties can significantly enhance the performance of perovskite solar cells by improving light harvesting and charge carrier dynamics.

## 2 Experimental Procedure

### 2.1 Materials

The materials used in the synthesis and fabrication processes included gadolinium oxide (Gd_2_O_3_, 99.9%), ytterbium oxide (Yb_2_O_3_, 99.9%), thulium oxide (Tm_2_O_3_, 99.9%), and erbium oxide (Er_2_O_3_, 99.9%) as lanthanide sources for the preparation of lanthanide trifluoroacetates (Ln­(TFA)_3_), all supplied by Sigma-Aldrich (Merck KGaA). Trifluoroacetic acid (CF_3_COOH, ≥99.0%), sodium hydroxide (NaOH, ≥97.0%), octadecene (ODE, 90.0%), oleic acid (OA, 90.0%), oleylamine (OM, 70%), ethanol (≥95.0%), cyclohexane (≥99.0%), distilled water, and toluene (≥99.9%) were also used. Indium tin oxide (ITO)-coated glass substrates (XY15S, 15 Ω/sq) from Xinyan Technology Ltd. and 99.99% purity nickel oxide (NiOx) sputtering targets from Super Conductor Materials, Inc., were used for PSC fabrication. Methylammonium iodide (MAI, ≥99.9%), lead iodide (PbI_2_, ≥99.999%), dimethylformamide (DMF, ≥99.8%), dimethyl sulfoxide (DMSO, ≥99.9%), bathocuproine (BCP, ≥99.99%), and chlorobenzene (≥99.0%) were supplied by Sigma-Aldrich (Merck KGaA). Phenyl-C61-butyric acid methyl ester (PCBM, ≥99.0%) was obtained from Ossila, and silver (Ag) was used for back contact deposition.

The concentration of Eu^3+^ was selected to optimize the balance between luminescence intensity and concentration quenching, ensuring enhanced brightness without significantly compromising the absorption and emission properties.

### 2.2 Nanoparticle Synthesis

The NaGdF_4_:18%Yb^3+^,2%Tm^3+^(Er^3+^)@NaGdF_4_:30%Eu^3+^ nanoparticles were synthesized using lanthanide (Ln^3+^) and sodium trifluoroacetates (Na­(TFA)) as precursors, obtained by dissolving the corresponding lanthanide oxides (Ln_2_O_3_) in trifluoroacetic acid (1.0 g in 32 mL of 1:1 H_2_O:TFA mixture). The synthesis was carried out in octadecene, oleic acid, and oleylamine, using 1.5 mmol of Ln­(TFA)_3_ and 1.5 mmol of Na­(TFA) in 10.7 mL ODE, 5.25 mL OA, and 7.05 mL OM, with an initial heating step at 100 °C under vacuum for 30 min to remove impurities and allow the formation of lanthanide complexes. This was followed by heating to 310 °C under an argon atmosphere for 20 min, leading to the formation of α-NaGdF_4_. The conversion to β-NaGdF_4_ was facilitated by rapidly injecting an excess Na­(TFA) solution (2.6 mmol Na­(TFA) in 7.1 mL ODE and 7.1 mL OA), followed by heating at 330 °C for 15 min. The NPs were then washed, precipitated with ethanol, and centrifuged at 8000 rpm for 10 min. The washing process was repeated with ethanol, cyclohexane, and water, and the particles were dried at 80 °C. Doping with Yb^3+^/Tm^3+^ or Yb^3+^/Er^3+^ was achieved by adjusting the amounts of Gd­(TFA)_3_ (1.2 mmol), Yb­(TFA)_3_ (0.27 mmol), and Tm­(TFA)_3_/Er­(TFA)_3_ (0.03 mmol). For core–shell NPs, the temperature was reduced to 260 °C before adding the shell precursors, consisting of 0.5 mmol of Gd­(TFA)_3_ or Y­(TFA)_3_ and 0.5 mmol of Na­(TFA), dissolved in 3.5 mL of ODE and 3.5 mL of OA. In Eu^3+^-doped shells, part of the Gd­(TFA)_3_ was replaced with 0.15 mmol of Eu­(TFA)_3_. This method, along with the subsequent approach used for nanoparticle synthesis, follows the procedure described by Rodrigues et al.^18^ and Calado et al.^19^

### 2.3 Device Fabrication

Perovskite solar cells (PSCs) were fabricated with a glass/ITO/NiO_*x*_/NPs/MAPbI_3_/PCBM/BCP/Ag architecture, as previously reported.^20–22^ NiO_*x*_ films (40 nm) were deposited by RF magnetron sputtering (150 W, 4 × 10^–3^ mbar Ar). The MAPbI_3_ precursor solution (1.2 M) was prepared by dissolving equimolar amounts of MAI and PbI_2_ in a 9:1 (v/v) DMF:DMSO mixture. The solution was stirred at 60 °C for 4 h, equilibrated overnight, and filtered using a 0.22 μm PTFE syringe filter. PCBM and BCP solutions were prepared at concentrations of 20 mg/mL in chlorobenzene and 1 mg/mL in ethanol, respectively. All solutions were handled in a nitrogen-filled glovebox. MAPbI_3_ films were deposited by spin-coating at 2,000 rpm for 10 s followed by 4,000 rpm for 20 s. A total of 90 μL of chlorobenzene was dripped 3 s after reaching 4,000 rpm. Films were then annealed at 125 °C for 10 min. PCBM was deposited dynamically by dispensing 60 μL of solution while spinning at 1,500 rpm for 15 s followed by 2,000 rpm for 20 s. The layer was then dried at 70 °C for 1 min. BCP was deposited by spin-coating 60 μL at 4,000 rpm for 40 s. Ag electrodes (150 nm) were deposited by thermal evaporation, defining an active area of 0.10 cm^2^. For NP-based devices, 250 μL of a 1 mg/mL nanoparticle dispersion in toluene was spin-coated onto the NiO_*x*_ layer at 2,000 rpm for 30 s, followed by annealing at 40 °C for 1 min. Each device substrate contained an array of 10 individual cells.

### 2.4 Characterization Techniques

X-ray diffraction (XRD) measurements were conducted using a Shimadzu XRD-6000 diffractometer, operated at 40 kV and 30 mA with Cu Kα radiation. Diffraction patterns were recorded in the angular range of 10° to 65°, with a step size of 0.02° and a scan speed of 2°/min. Slits of 1° were used for divergence and scattering, while a 0.30 mm slit was employed for reception.

A JEOL JEM-2100 was utilized for transmission electron microscopy (TEM) imaging, selected area electron diffraction (SAED), and energy dispersive X-ray spectroscopy (EDS). The system operates with a LaB_6_ electron source at 200 kV, achieving spatial resolutions of 0.25 nm in TEM mode and 0.2 nm in scanning transmission electron microscopy (STEM) mode. EDS mapping was performed to analyze the elemental distribution, while high-resolution bright-field STEM imaging and CMOS cameras (4k × 4k) captured detailed images and electron diffraction patterns of nanometric regions.

Emission and excitation spectra of the nanoparticle samples were measured using a FluoTime 300 spectrofluorometer (FT300, PicoQuant) with an additive emission monochromator featuring a grating with 1200 grooves mm^–1^ blazed at 500 nm (reciprocal linear dispersion of 1.4 nm mm^–1^). The system was coupled to a photomultiplier (PMA-C 192-N-M, PicoQuant) and employed the right-angle acquisition mode with a 300 W xenon arc lamp as the excitation source. Spectra were recorded at room temperature (298 K) with an integration time of 0.5 s and spectral resolution of 0.5 nm. The emission spectra were corrected for the spectrofluorometer’s detection and optical spectral response, while the excitation spectra were corrected for the lamp’s spectral intensity distribution using a photodiode reference detector. Upconverting emission spectra were measured with a 980 nm laser diode (LDH-P-C-980MB, PicoQuant) as the excitation source, operating at a power density of 62 W cm^–2^. Intensity decay profiles were recorded in the same equipment using a 10 W Xe flash lamp and the 980 nm laser in the pulsed mode as excitation modes, monitoring Eu^3+^ emission at 615 nm. The decay curves were adjusted to a monoexponential decay function *I*(*t*) = *I*_0_ + *A*·*e* ^–*t/τ*^, where *I*(*t*) is the photoluminescence intensity at the time *t*, *I*_0_ is the background intensity, τ is the emission lifetime, and *A* is the amplitude.

The quantum yield (QY) measurements were performed using a Hamamatsu C13534 system equipped with a 150 W xenon lamp as the excitation source. This system allows for precise absolute quantum yield determination by integrating the emitted light over all angles, eliminating the need for comparative standards. The setup includes an integrating sphere coated with a highly reflective material to ensure accurate light collection and a spectrometer for analyzing excitation and emission spectra.

The photovoltaic properties of solar cells were characterized using a VeraSol LSH-7520 LED solar simulator, classified as AAA according to IEC 60904-9 standards. The key photovoltaic parameters short-circuit current density (*J*_SC_), open-circuit voltage (*V*_OC_), fill factor (FF), and power conversion efficiency (PCE) were measured. Additionally, the simulator allowed measurements under varying light intensities, effectively simulating real-world operational conditions. Data acquisition and analysis were performed using LabView-based software, ensuring precision and streamlined workflows.

External quantum efficiency (EQE) measurements were carried out using a Newport QUANTX-300 system for precise spectral response characterization in the 320 to 1800 nm range. The measurements were conducted at room temperature with the system equipped with light biasing capabilities, electrical probing, and a lock-in amplifier for stable signal acquisition. The EQE measurements were specifically performed over the wavelength range of 350 to 800 nm.

## 3 Results and Discussion

The NaGdF_4_:Yb^3+^,Er^3+^ and NaGdF_4_:Yb^3+^,Tm^3+^ core NPs were synthesized via a thermolysis approach. Typical TEM micrographs of β-NaGdF_4_:Yb^3+^,Er^3+^ and β-NaGdF_4_:Yb^3+^,Tm^3+^ NPs reveal that both exhibit a similar quasi-spherical morphology, as illustrated in Figures S1a and S2a (Supporting Information). Furthermore, size distribution histograms derived from these micrographs (Figures S1b and S2b, Supporting Information) indicate average particle diameters of 12.0 ± 1.0 nm for β-NaGdF_4_:Yb^3+^,Er^3+^ and 12.5 ± 1.0 nm for β-NaGdF_4_:Yb^3+^,Tm^3+^, respectively.

To assess the effect of inert shell growth, the corresponding core–shell nanoparticles were analyzed, as shown in Figure 1b,c. A noticeable increase in particle size was observed after shell deposition: the Er^3+^ and Tm^3+^ based core–shell NPs reached average diameters of 19.0 ± 1.0 nm (ERN) and 16.0 ± 1.0 nm (TMN), respectively. These values correspond to estimated shell thicknesses of approximately 3.5 nm for ERN and 1.8 nm for TMN, confirming successful and uniform shell growth in both systems, with a more substantial shell observed in the Er^3+^-doped nanoparticles.

**Figure 1:**
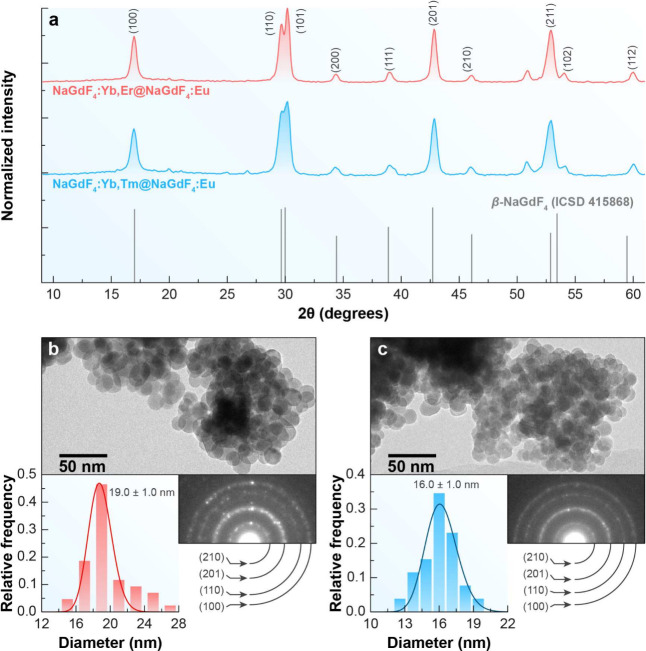
Structural and morphological characterization of β-NaGdF_4_-based NPs. (a) XRD patterns confirm the hexagonal β-phase for ERN (red) and TMN (blue), matching the ICSD 415868 reference. (b, c) TEM images show well-dispersed NPs with average diameters of 19 ± 1 nm for ERN (b) and 16 ± 1 nm for TMN (c). Insets: particle size distributions and SAED patterns confirming crystallinity.

Figures S1c and S2c (Supporting Information) present the XRD patterns of both cores, which exhibit diffraction peaks corresponding to a hexagonal crystal structure (space group *P*6_3_/*m*), as referenced in the ICSD 415868 file card. These patterns were indexed to the crystallographic planes (100), (110), (101), (200), (111), (201), (211), (102), and (112), confirming the β-phase of the NaGdF_4_:Yb^3+^,Er^3+^ and NaGdF_4_:Yb^3+^,Tm^3+^ NPs. The results are consistent with previous reports in the literature.^18,19^

The photoluminescence spectra in the visible region, acquired from all NPs under 980 nm excitation (62 W cm^–2^), are shown in Figures S1d and S2d (Supporting Information). The emission process originates from the excitation of Yb^3+^ ions, attributed to the transition from ^2^F_7/2_ to ^2^F_5/2_. This is followed by energy transfer to Tm^3+^ and Er^3+^ ions,^23^ albeit through distinct pathways. In the NaGdF_4_:Yb^3+^,Er^3+^ NPs (Figure S1d), characteristic emission lines were observed, corresponding to the transitions ^2^H_9/2_ → ^4^I_15/2_ (408 nm), ^2^H_11/2_ → ^4^I_15/2_ (528 nm), ^4^S_3/2_ → ^4^I_15/2_ (550 nm), ^4^F_9/2_ → ^4^I_15/2_ (661 nm) and ^2^H_9/2_ → ^4^I_11/2_ (698 nm) consistent with previous reports.^12^ Conversely, the NaGdF_4_:Yb^3+^,Tm^3+^ NPs (Figure S2d) exhibited typical emission bands from the transitions ^1^G_4_ → ^3^H_6_ (476 nm), ^1^G_4_ → ^3^F_4_ (645 nm), ^3^F_3_ → ^3^H_6_ (696 nm) and ^3^H_4_ → ^3^H_6_ (800 nm), as documented in the literature.^24^ These results confirm the upconversion properties of the core NPs.

Figure 1 illustrates the structural and morphological characteristics of core–shell β-NaGdF_4_-based NPs doped with Yb^3+^/Er^3+^ (ERN) and Yb^3+^/Tm^3+^ (TMN), coated with a Eu^3+^-doped shell. The XRD patterns shown in Figure 1a confirm the presence of the hexagonal β-phase in both samples, with peaks aligning to the reference from ICSD 415868 and indexed to the characteristic planes (100), (110), (101), among others.^25^ The particles display a monodisperse size distribution, as indicated by the minimal error in the average diameters obtained from TEM images (Figure 1b,c). The histograms show average diameters of 19.0 ± 1.0 nm for ERN and 16.0 ± 1.0 nm for TMN, corresponding to a NaGdF_4_:Eu^3+^ shell thickness of 3.5 and 1.8 nm, respectively. Suggesting that adding a shell of NaGdF_4_:Eu^3+^ to the core increased the overall size of NPs in a few nanometers. Furthermore, the SAED patterns corroborate the XRD data, reinforcing the hexagonal crystalline structure by indexing the rings to the defining lattice planes of β-NaGdF_4_. These findings confirm the successful synthesis of highly crystalline, monodisperse NPs with controlled size and high phase purity.

The synthesized ERN demonstrates upconversion emission from the well-established energy transfer between Yb^3+^ and Er^3+^. The emission bands observed correspond to the transitions ^2^H_9/2_ → ^4^I_15/2_ (408 nm), ^2^H_11/2_ → ^4^I_15/2_ (520 nm), ^4^S_3/2_ → ^4^I_15/2_ (540 nm), ^2^H_9/2_ → ^4^I_13/2_ (556 nm), ^4^F_9/2_ → ^4^I_15/2_ (654 nm), and ^2^H_9/2_ → ^4^I_11/2_ (698 nm) of Er^3+^, upon excitation with a 980 nm continuous-wave laser diode (Figure 2a).^26–28^ Moreover, emission bands corresponding to the ^5^D_0_ → ^7^F_1_ and ^5^D_0_ → ^7^F_2_ transitions of Eu^3+^ were also detected, peaking at 590 and 615 nm, respectively. It is crucial to note that, despite these Eu^3+^ emission bands being excited by 980 nm radiation, Eu^3+^ ions do not absorb near-infrared photons. Consequently, the observed emissions must originate from the energy transfer between Er^3+^ and Eu^3+^.^29,30^

**Figure 2:**
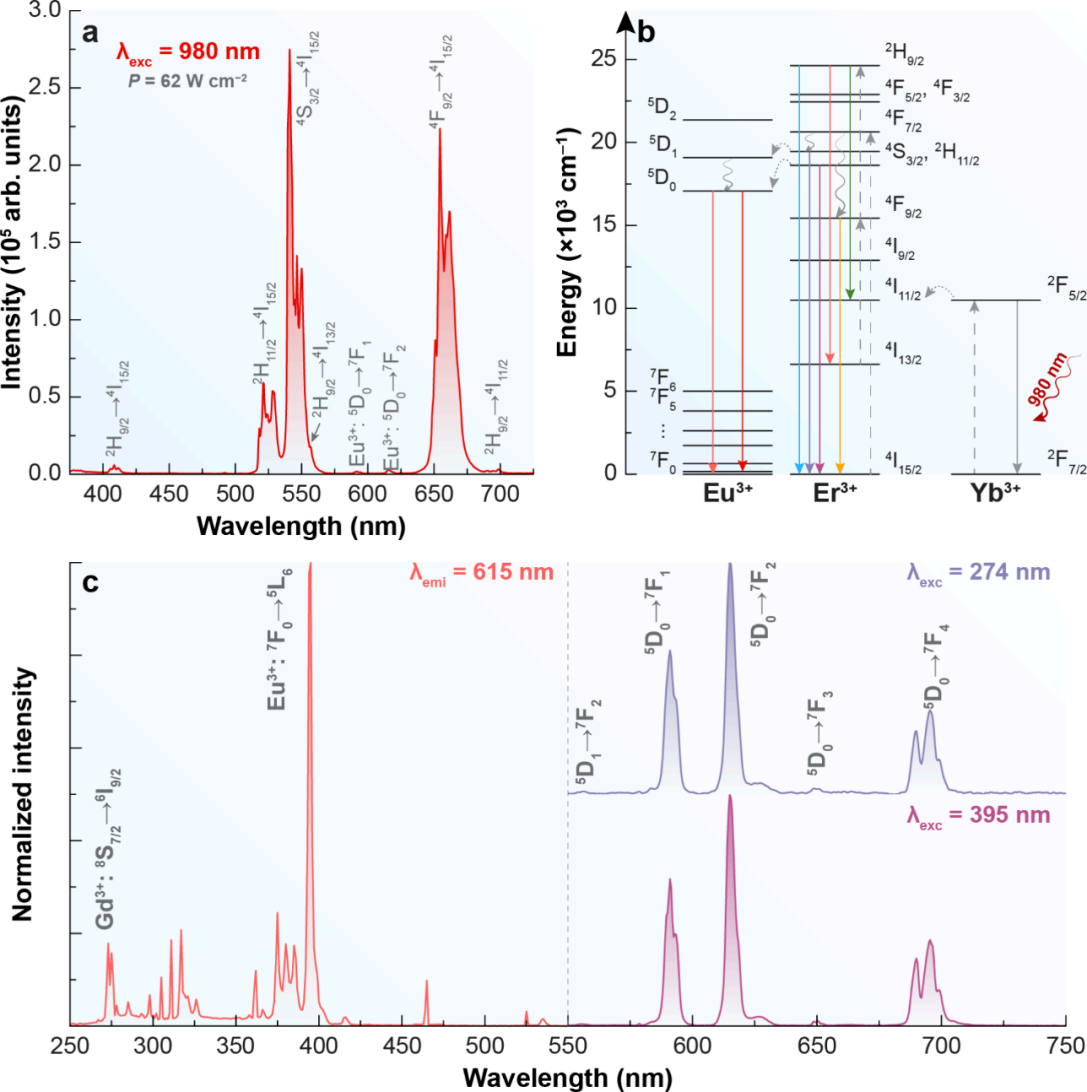
Optical characterization of ERN (Er^3+^). (a) Upconverting emission spectra under 980 nm. (b) Partial energy level diagram depicting the energy transfer pathway between Yb^3+^, Er^3+^, and Eu^3+^. The solid lines indicate radiative transitions; curly arrows display nonradiative decays, and dotted lines represent nonradiative energy transfer. (c) Excitation spectrum monitoring the emission at 615 nm (left) and downshifting emission spectra under 274 and 395 nm excitation (right).

When the energy gap between the ^4^S_3/2_ (Er^3+^) and ^5^D_0_ (Eu^3+^) levels is approximately 1,000 cm^–1^, and the ^2^H_11/2_ (Er^3+^) and ^5^D_1_ (Eu^3+^) energy levels are nearly overlapping, energy transfer from Er^3+^ to Eu^3+^ becomes feasible,^31^ as illustrated in Figure 2b. Moreover, both the emission profile and the lifetime of Eu^3+^ remain constant, regardless of whether direct excitation at 395 nm or Yb^3+^-mediated excitation at 980 nm is employed, as observed in Figure 3a,b.

**Figure 3:**
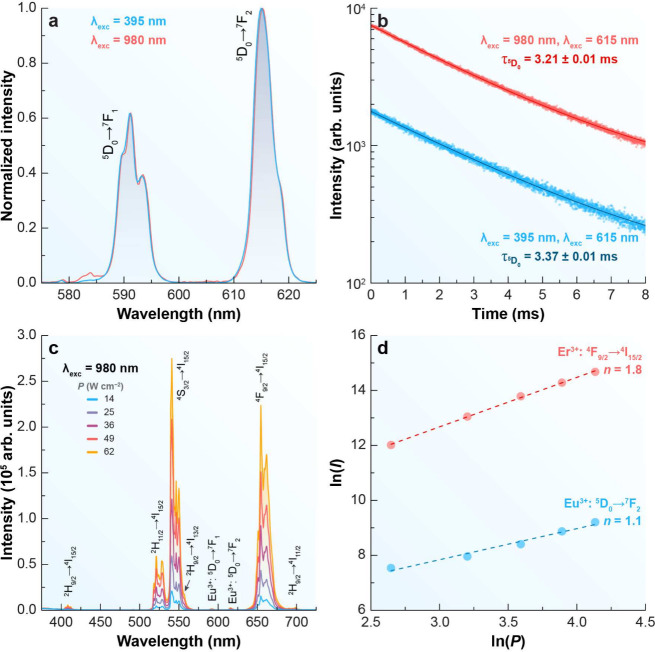
Optical characterization of ERN (Eu^3+^). (a) Emission spectra of the ERN under excitation at 395 nm (Eu^3+^, blue) and 980 nm (Er^3+^, red). (b) Emission decay profiles of the ^5^D_0_ emitting level of Eu^3+^ under excitation at 395 and 980 nm, monitoring the emission at 615 nm. The symbols represent the experimental data, and the solid lines correspond to the best monoexponential fitted curves with the calculated ^5^D_0_ lifetime (τ_^5^ *D*_0__). (c) Upconverting emission spectra of ERN under 980 nm excitation at varying excitation power *P*. (d) Double logarithmic plot of the integrated intensities *I* of the ^5^D_0_ → ^7^F_2_ (Eu^3+^, 609–622 nm) and ^4^F_9/2_ → ^4^I_15/2_ (Er^3+^, 635–685 nm) emission bands from panel (c), indicating the corresponding number of photons *n*.

A straightforward approach to demonstrate upconversion in materials doped with Yb/Er or Yb/Tm ion pairs is by analyzing the power (*P*) dependence of the emitted intensity (*I*). Because upconversion is a nonlinear process, the emission follows a power law, *I* ∝ *P* ^*n*^, where *n* corresponds to the number of absorbed photons.^32^ For the ERN sample, the Er^3+^ emission (^4^ *F*_9/2_ → ^4^ *I*_15/2_, peaking at 654 nm) displays *n* = 1.8, confirming that upconversion is indeed the mechanism behind Er^3+^ emission. In contrast, the Eu^3+^ emission (^5^ *D*_0_ → ^7^ *F*_2_, peaking at 615 nm) observed under 980 nm excitation shows *n* = 1.1 (Figure 3c,d). Although this slope is close to unity, the process must still be regarded as upconversion, since NIR excitation produces visible Eu^3+^ emission. The low slope value reflects that the emission originates from Er^3+^ →Eu^3+^ energy transfer rather than from direct multiphoton absorption by Eu^3+^ ions. This interpretation is further supported by the emission lifetimes and spectral profiles recorded under 395 and 980 nm excitation (Figure 3a,b), which are nearly identical, confirming that the observed signal originates from Eu^3+^ emission. Regardless of the excitation pathway, the same emission profile (peak positions and full width at half-maximum) is observed, giving rise to characteristic Eu^3+^ emission bands, including ^5^D_1_ → ^7^F_2_ (556 nm) and ^5^D_0_ → ^7^F_*J*=1–4_ (590, 615, 650, 696 nm).^33^ While both upconversion and downshifting processes have been identified for TMN, no evidence of energy transfer from Tm^3+^ to Eu^3+^ was observed (Figure 4).

**Figure 4:**
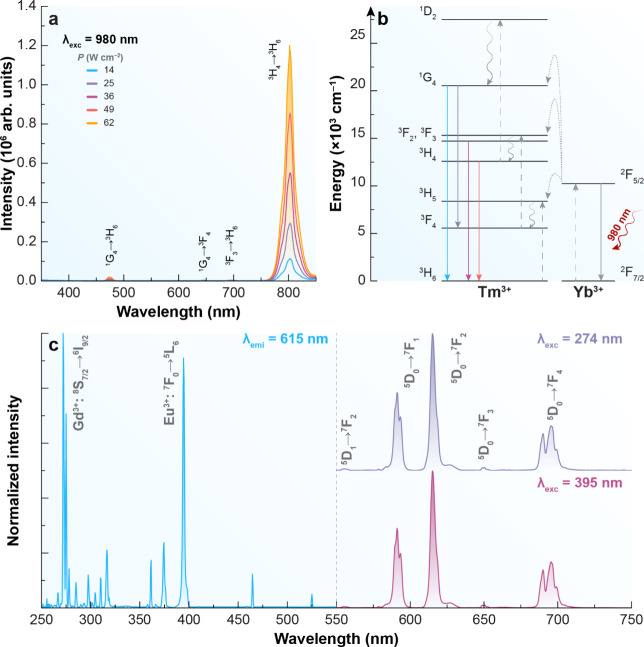
Optical characterization of TMN (Tm^3+^). (a) Upconverting emission spectra under 980 nm excitation at varying power densities, *P*. (b) Partial energy level diagram depicting the energy transfer pathway between Yb^3+^ and Tm^3+^. The solid lines indicate radiative transitions; curly arrows display nonradiative decays, and dotted lines represent nonradiative energy transfer. (c) Excitation spectrum monitoring the emission at 615 nm (left) and downshifting emission spectra under 274 and 395 nm excitation (right).

Eu^3+^ emission can be activated under different excitation pathways. Under NIR excitation (980 nm), it arises from Er^3+^-mediated upconversion, while under UV excitation it can result either from direct Eu^3+^ absorption (^7^F_0_ → ^5^L_6_, 395 nm) or from Gd^3+^-mediated transfer (^8^S_7/2_ → ^6^I_9/2_, 274 nm),^34^ as illustrated in Figure 2c.

In short, Eu^3+^ emission in the ERN sample arises from a dual mechanism:Er^3+^-triggered upconversion under NIR excitation and direct/indirect excitation (via Eu^3+^ or Gd^3+^) under UV excitation. This combination enables efficient harvesting of both UV and NIR regions of the solar spectrum, which represents a significant advantage for enhancing solar cell performance.

In this study, upconversion was considered as a complementary mechanism to downshifting, aiming to broaden the absorption spectrum of perovskite solar cells (PSCs) and potentially enhance their efficiency. Downshifting alone converts high-energy photons into wavelengths more efficiently absorbed by the perovskite layer, whereas upconversion enables sub-bandgap photons in the NIR to be converted into higher-energy photons usable by the active layer. This synergistic combination allows for more comprehensive harvesting of the solar spectrum. Although previous studies have shown that core/shell architectures can improve downshifting photoluminescence quantum yield (PLQY) through surface passivation, the primary goal of the present work was to explore the role of these multiple luminescent properties in potentially enhancing PSC performance.

Table 1 presents the quantum yield (QY) values for different excitations for core and core–shell architecture NPs, highlighting the best result obtained for the ERN sample in powder form, with a QY of 16.41% when excited at 395 nm in Eu^3+^. This high value reflects the efficiency of downshifting processes, which enhance light absorption in the visible region.^35^ Only the core–shell nanoparticles were incorporated into PSCs, as they exhibited significantly higher photoluminescence quantum yields (PLQY) compared to their core-only counterparts under relevant excitation conditions (Table 1). For instance, the PLQY of ERN increased nearly 300-fold, from 0.0001% (core) to 0.0291% (core–shell) under 980 nm excitation at 1862.6 W cm^–2^. This improvement is attributed to the passivating effect of the shell, which mitigates nonradiative recombination by shielding Yb^3+^ and Er^3+^ ions at the nanoparticle surface and reducing surface defect density.^36^

**Table 1:** Quantum Yield (QY) Values of ERN and TMN Samples under Different Excitation Conditions and Power Densities

Samples	Excitation (nm)	Power density (W cm^–2^)	QY (%)
ERN (core)	980	944.2	0.0001
	980	1862.6	0.0019
ERN (core–shell)	274		7.7600
	395		16.4100
	980	944.2	0.0001
	980	1862.6	0.0291
TMN (core)	980	944.2	0.3500
TMN (core–shell)	274		5.1300
	395		7.8300
	980	944.2	0.0240

Although steady-state emission intensities measured under 980 nm excitation remained similar between core and core–shell samples (Figure S3, Supporting Information), the substantial enhancement in PLQY confirms a more efficient radiative process in the core–shell structure.

In contrast, the TMN nanoparticles exhibited a decrease in PLQY upon shell growth (from 0.35% to 0.024% at 944.2 W cm^–2^), indicating that the shell may have introduced additional nonradiative pathways or hindered energy transfer. Nonetheless, the TMN core–shell sample showed strong emission under UV excitation (274 and 395 nm), which is relevant for downshifting (Figure 4). Therefore, despite the differing behaviors, the core–shell architecture was preferred for device integration due to its superior PLQY and luminescent performance under practical excitation conditions.

Figure 5a shows *J*–*V* curves for the control and ERN-bearing devices, highlighting the significant increase in power conversion efficiency in the ERN device (14.50%) compared to the control (11.90%). The device architecture in the inset features the glass/ITO/NiO_*x*_/NPs/Perovskite/PCBM/BCP/Ag layers optimized to maximize charge carrier extraction and transport. The superior performance of the ERN device can be attributed to two main factors: (i) the high QY of 16.41% obtained for excitation at 395 nm, reflecting efficient downshifting processes,^37,38^ and (ii) the passivation effect provided by the ERN NPs. Passivation reduces structural defects, minimizing nonradiative recombination of charge carriers.^39–41^ The ERN NPs are also expected to enhance light scattering within the cell, contributing to more effective optical absorption.The enhanced performance of the ERN-based device is primarily attributed to the efficient downshifting emission of the nanoparticles, as demonstrated by the relatively high photoluminescence quantum yield (PLQY) of 16.41% under 395 nm excitation.^37,38^ This downshifting process improves the spectral matching with the perovskite absorption, contributing to increased photon harvesting. Additionally, the ERN nanoparticles may promote light scattering within the device, enhancing optical path lengths and further improving light absorption. Although a passivation effect is often considered beneficial in nanostructured materials,^39–41^ in this case, its contribution to device performance is expected to be limited, given the low upconversion PLQY (<0.1%), which reduces its direct impact under operational conditions.

**Figure 5:**
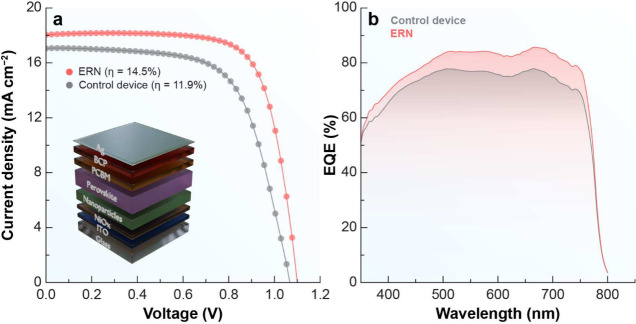
Electrical and optoelectrical characterizations of the PSCs. (a) *J*–*V* curves of the best solar cells of the photovoltaic prototype, after incorporating ERN, compared to those of a control device. The inset shows the architecture of the fabricated prototype. (b) The EQE spectrum of the best solar cells of the device containing ERN compared with that of the control device.

External quantum efficiency spectra are presented in Figure 5b. It can be observed that the ERN device consistently shows higher EQE values across the analyzed spectral range, particularly in the visible region, where the ERN NPs promote more efficient conversion of incident light. These results support the improvement in photogenerated charge extraction, driven by defect passivation and optimization of the interfaces between the device layers.

Interestingly, while the high QY at 395 nm indicates greater efficiency in photon absorption and reemission in the visible region, the difference in EQE suggests additional contributions to the increase in PCE beyond the optical gain provided by downshifting. The device architecture, which integrates the NPs at the interface between NiO_*x*_ and the perovskite layer, plays a crucial role in this process, maximizing charge carrier collection and reducing losses associated with nonradiative recombination.

Figure 6 illustrates the effect of incorporating ERN on the photovoltaic parameters of ten perovskite solar cells within a photovoltaic device, including the cell with the highest PCE depicted in Figure 5. The parameters analyzed are open-circuit voltage (Figure 6a, *V*_OC_), short-circuit current density (Figure 6b, *J*_SC_), fill factor (Figure 6c, FF), and power conversion efficiency (Figure 6d, PCE). Each graph compares cells containing ERN with control cells, revealing significant improvements in the performance of the ERN-containing cells over their controls.

**Figure 6:**
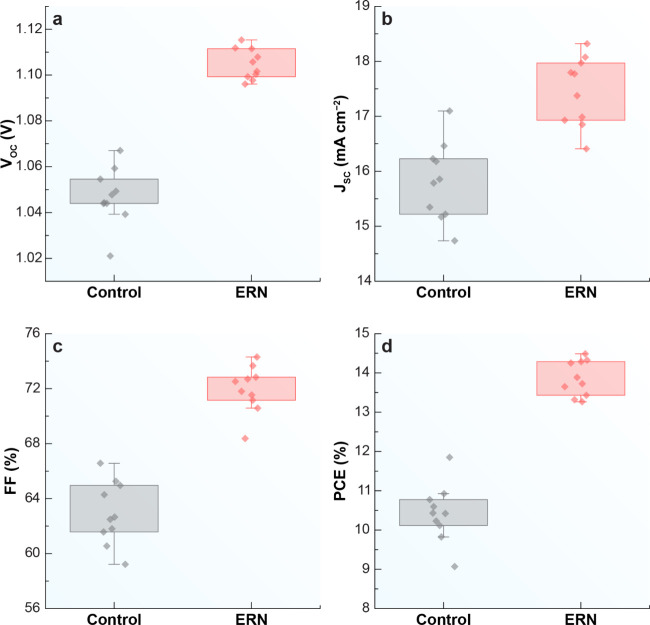
Photovoltaic performance of PSCs with (ERN) and without (Control) the addition of ERN. (a) Open-circuit voltage (*V*_OC_), (b) short-circuit current density (*J*_SC_), (c) fill factor (FF), and (d) power conversion efficiency (PCE).

The observed increase in *V*_OC_ is likely a result of enhanced charge separation or reduced recombination losses at the interface.^42^ These findings suggest ERN NPs effectively act as a passivation layer or promote beneficial electronic interactions with the perovskite material.^43^ The improvement in *J*_SC_ values can be attributed to enhanced light absorption through two distinct mechanisms: (i) efficient light conversion facilitated by the downshifting properties of the NPs, which expand the absorption spectrum,^37,38^ and (ii) increased light scattering within the device, which leads to a more effective utilization of the incident light. Additionally, more efficient charge transport and reduced nonradiative recombination losses^39–41^ also contribute to the observed performance improvement. Additionally, the rise in FF suggests that incorporating ERN into the cells reduces series resistance or enhances interfacial contact between device layers, facilitating more effective charge extraction and transport.^44^ The increase in PCE is a consequence of the combined enhancements in *V*_OC_, *J*_SC_, and FF, implying that ERN NPs contribute to improving overall device performance through potential synergistic effects on light harvesting, charge carrier dynamics, and interface passivation.^45,46^

The photovoltaic performance of the PSCs with and without TMN incorporation is summarized in Figure 7. The power conversion efficiency (PCE) of the best-performing devices increased from 13.0% for the control device to 14.7% for the TMN-based device, as shown in Figure 8a. Despite this improvement in PCE, no significant changes were observed in the open-circuit voltage (*V*_OC_) or fill factor (FF), suggesting that the performance enhancement is primarily attributed to increased current generation or improved charge transport.

**Figure 7:**
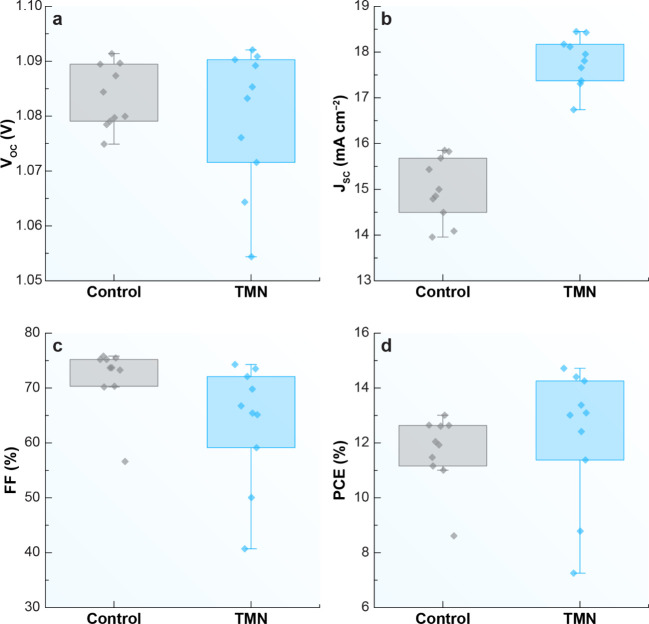
Photovoltaic performance of PSCs with (TMN) and without (Control) the addition of TMN. (a) Open-circuit voltage (*V*_OC_), (b) short-circuit current density (*J*_SC_), (c) fill factor (FF), and (d) power conversion efficiency (PCE).

**Figure 8:**
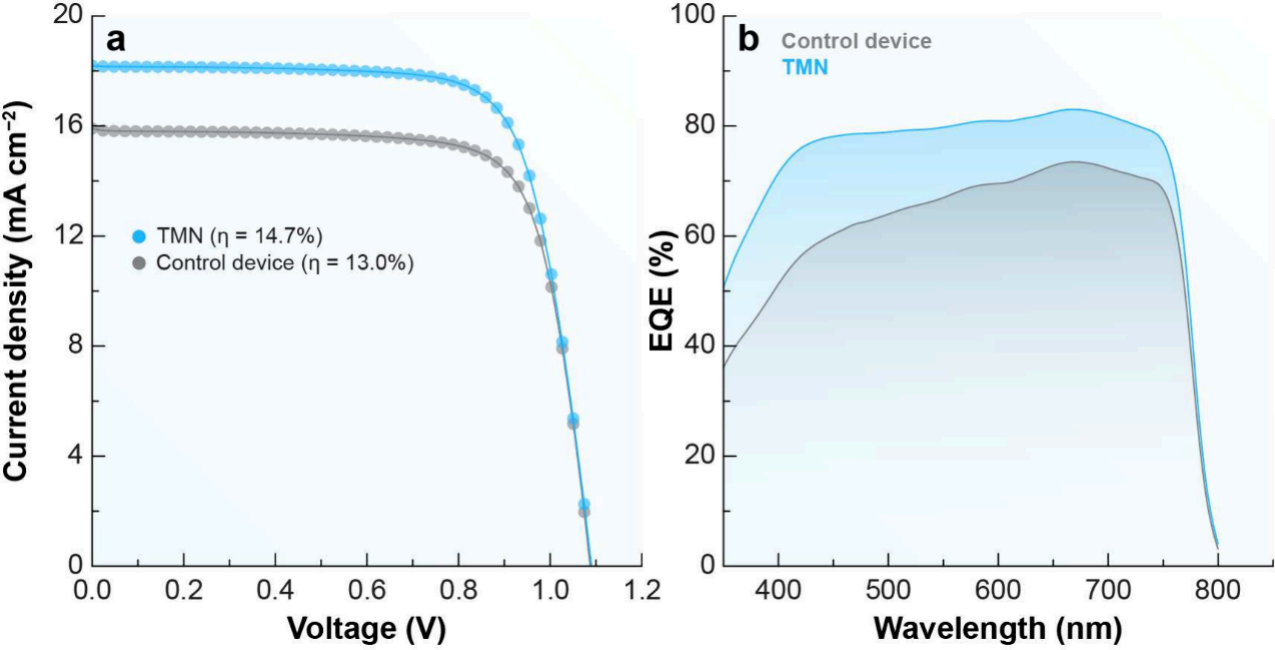
Electrical and optoelectrical characterizations of the PSCs. (a) *J*–*V* curves of the best solar cells of the photovoltaic prototype, after incorporating TMN, compared those of a control device. (b) The EQE spectrum of the best solar cells of the device containing TMN compared with that of the control device.

These findings are consistent with the *J*–*V* curves presented in Figure 8a, where the TMN-based device exhibits a slightly higher current density compared to the control. Furthermore, the external quantum efficiency (EQE) spectra shown in Figure 8b indicate a broader and more efficient photon harvesting in the TMN-incorporated devices, particularly across the visible region. This enhanced light absorption likely contributes to the observed increase in PCE.

In contrast, ERN-based PSCs exhibited superior performance across all key photovoltaic parameters. This enhancement is mainly due to the higher photoluminescence quantum yield (PLQY) of the Eu^3+^-doped shell under 395 nm excitation, which significantly boosts downshifting efficiency by converting high-energy photons into wavelengths better absorbed by the perovskite. Moreover, the thicker shell in ERN nanoparticles (3.5 nm vs 1.8 nm for TMN) not only improves surface passivation, effectively reducing nonradiative recombination losses, but also stabilizes the Eu^3+^ ions responsible for downshifting emission. The synergistic effect of enhanced photon conversion and reduced recombination results in the notable improvement of photovoltaic performance observed in ERN-containing devices.

## 4 Conclusions

The incorporation of ERN and TMN NPs in PSC devices has significantly influenced their photovoltaic performance. Although these nanoparticles are known for their upconversion and downshifting optical properties, the contribution of upconversion-capable NPs to photovoltaic efficiency remains to be confirmed. Further investigation is needed to determine the extent of their beneficial effects. Additionally, these NPs have broadened the spectral response of PSCs, enhancing light absorption and potentially improving overall device efficiency. Specifically, PSCs containing ERN exhibited a 22.19% increase in power conversion efficiency, achieving 14.50% compared to the control cells, which reached 11.90%. This improvement can be attributed to the high efficiency of the downshifting process of the ERN NPs, which demonstrated an absolute quantum yield of 16.41% when excited at 395 nm. Additionally, morphological aspects may have played a crucial role: ERN NPs possess a thicker shell (3.5 nm) compared to TMN (1.8 nm), which can offer more effective surface passivation and reduce nonradiative recombination, thereby further enhancing device performance.

The presence of TMN also contributed to an increase in power conversion efficiency, though the relative improvement was more modest at 13.23%. This more limited enhancement may be related not only to differences in optical properties, but also to the thinner shell structure of TMN NPs, which may result in less efficient surface passivation.

Moreover, key photovoltaic parameters, including open-circuit voltage, short-circuit current density, and fill factor, were enhanced due to the addition of the NPs. This enhancement may be linked to the passivation of interfaces and a reduction in nonradiative recombination losses. However, it remains unclear whether these enhancements are primarily related to the photonic properties of the NPs, their structural attributes, the interplay of multiple factors, or other mechanisms, such as solvent effects and surface passivation. Further research is necessary to determine if the improvements stem from photophysical processes, such as heightened photon conversion or modifications in device architecture. Additionally, the long-term stability of the cells and the investigation of new doping compositions warrant exploration in future studies to assess the ongoing advancement of photovoltaic technologies.

## Supplementary Material



## Notes

TEM images, size distribution histograms, XRD patterns, and photoluminescence spectra for NaGdF_4_:Yb^3+^,Er^3+^ and NaGdF_4_:Yb^3+^,Tm^3+^ core nanoparticles; emission spectra comparing core and core–shell structures NaGdF_4_:Yb^3+^,Er^3+^@NaGdF_4_:Eu^3+^ and NaGdF_4_:Yb^3+^,Tm^3+^@NaGdF_4_:Eu^3+^ (PDF)
