# Supplementary material for: Dual Spectral Matching in Perovskite Solar Cells via Upconverting plus Downshifting Nanoparticles
Source: ACS Appl Energy Mater. 2025 Sep 30;8(19):14881–92. doi: 10.1021/acsaem.5c02611 (PMC12522088; doi:10.1021/acsaem.5c02611)
Supplement: Supplementary file 1 [file ae5c02611_si_001.pdf]

Supporting Information

Dual Spectral Matching in Perovskite Solar Cells via Upconverting plus Downshifting Nanoparticles

Milliane P. S. Palácio*[1], Luis P. M. dos Santos*[1], Leonardo C. E. Barros[2], Nagyla Oliveira[3], Sergio F. N. Coelho[3], Edgar A. C. Coimbra[4], Daniel P. Camilo[4], F. Anderson S. Lima[5], Fernando E. Maturi[6], Ugur D. Menda[4], Fernando A. Sigoli*[3], Wagner F. Silva[2], Carlos Jacinto[2], Paulo Andre[7], Manuel J. Mendes[4], Rute A. S. Ferreira[6], and Igor F. Vasconcelos*[1]

[1]Department of Metallurgical and Materials Engineering, Federal University of Ceará, Fortaleza, Ceará 60455-760, Brazil
[2]Department of Physics, Federal University of Alagoas, Maceió, Alagoas 57072-900, Brazil
[3]Department of Inorganic Chemistry, State University of Campinas, Campinas, São Paulo 13083-970, Brazil
[4]i3N/CENIMAT, Department of Materials Science, NOVA School of Science and Technology and CEMOP/UNINOVA, Campus de Caparica, Caparica 2829-516, Portugal
[5]Center of Technology, University of Fortaleza, Fortaleza, Ceará 60811-905, Brazil
[6]Department of Physics and CICECO - Aveiro Institute of Materials, University of Aveiro, Aveiro 3810-193, Portugal
[7]Department of Electrical and Computer Engineering and Instituto de Telecomunicações, Instituto Superior Técnico, Universidade de Lisboa, Lisbon 1049-001, Portugal

*Corresponding author: millianepassos@alu.ufc.br, santoslpm@metalmat.ufc.br, fsigoli@unicamp.br

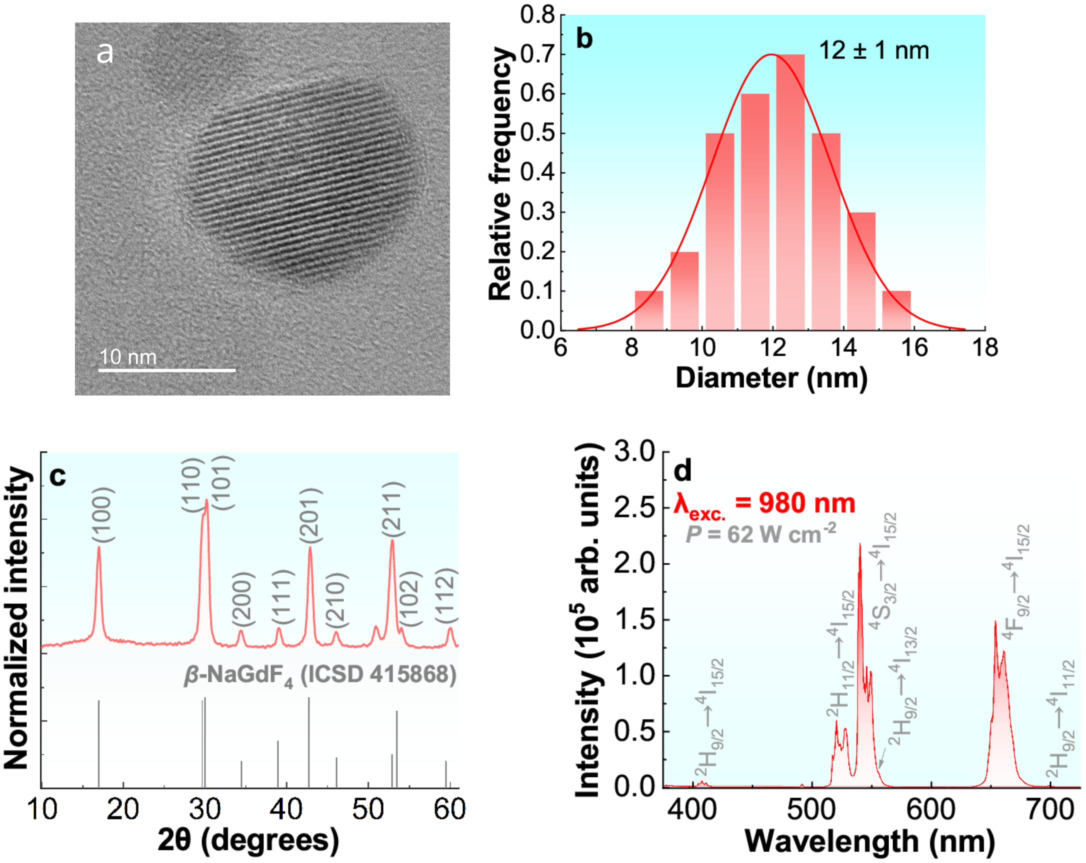

Figure S1: Characterization of the $NaGdF_4$:Yb,Er core: (a) TEM image, (b) size distribution from TEM with an average size of approximately 12 nm, (c) XRD pattern, and (d) photoluminescence spectrum.

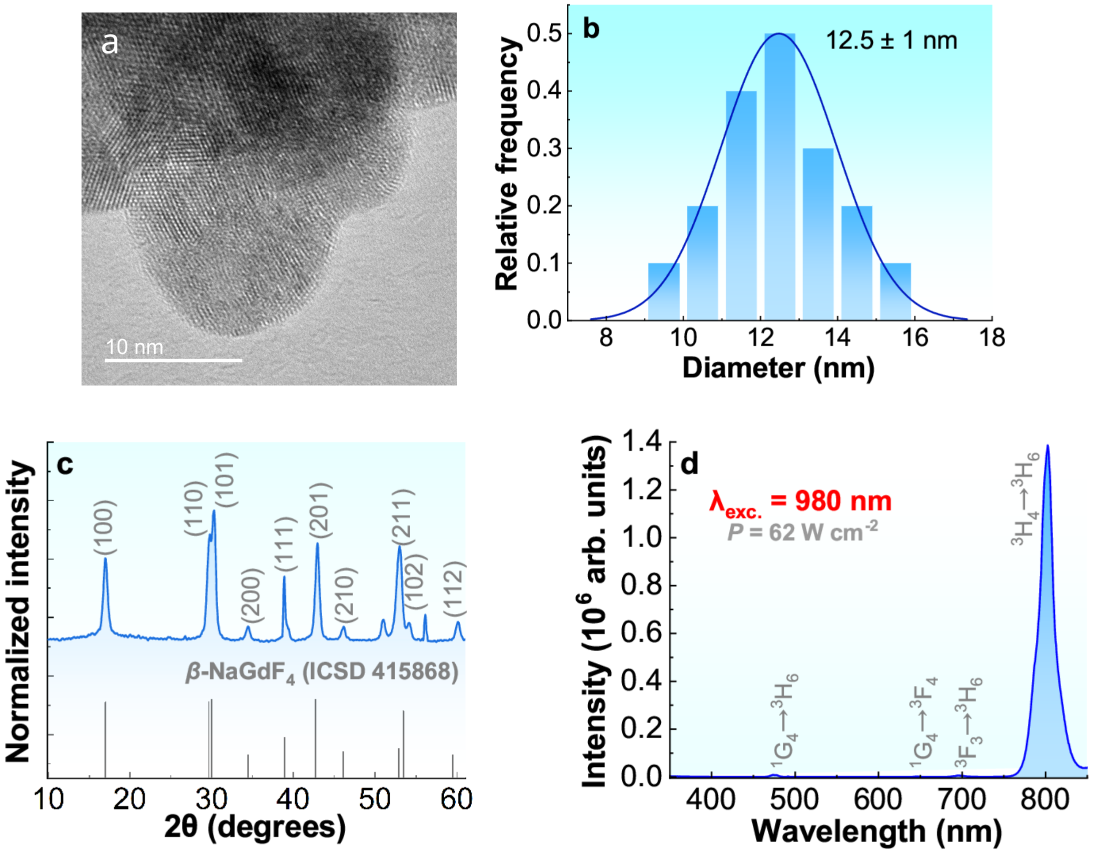

Figure S2: Characterization of the $NaGdF_4$:Yb,Tm core: (a) TEM image, (b) size distribution from TEM with an average size of approximately 12.5 nm, (c) XRD pattern, and (d) photoluminescence spectrum.

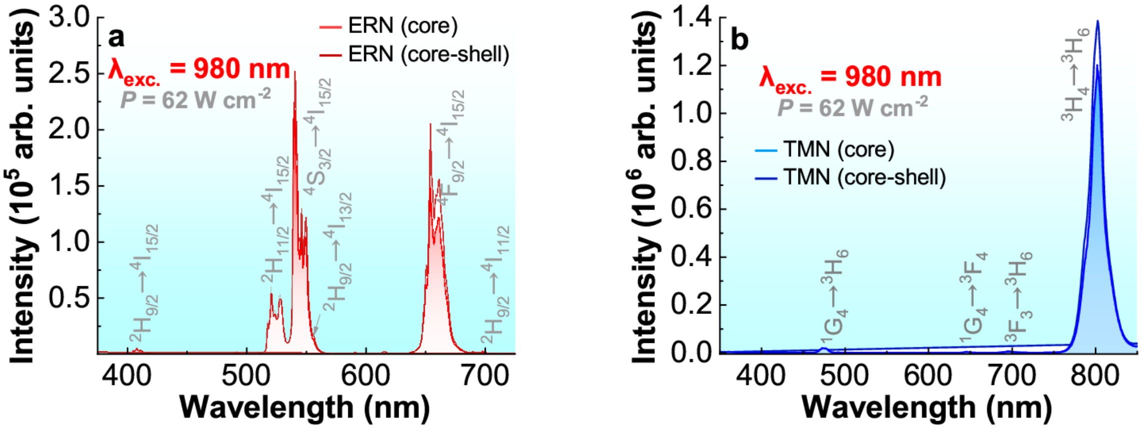

Figure S3: Emission of: (a) ERN (core): $NaGdF_4$:Yb,Er and ERN (core-shell): $NaGdF_4$:Yb,Er@$NaGdF_4$:Eu, (b) TMN (core): $NaGdF_4$:Yb,Tm and TMN (core-shell): $NaGdF_4$:Yb,Tm@$NaGdF_4$:Eu.
